# Bio-process performance, evaluation of enzyme and non-enzyme mediated composting of vegetable market complex waste

**DOI:** 10.1038/s41598-020-75766-3

**Published:** 2020-11-13

**Authors:** V. Murugesan, D. Joshua Amarnath

**Affiliations:** 1 Sathyabama Institute of Science and Technology, Chennai, 600119 India; 2 Department of Chemical Engineering, Sathyabama Institute of Science and Technology, Chennai, 600119 India

**Keywords:** Environmental biotechnology, Applied microbiology, Bacteriology, Environmental microbiology, Industrial microbiology, Biological techniques, Biotechnology, Microbiology

## Abstract

Vegetable Market have become major sources of organic waste. Some of such waste when being diverted to landfills not only increase the landfill loading but also contribute to increase greenhouse gas emission. Of the many technologies available in handling such hugely generated waste, composting has proven very effective for decades. Enzyme and non-enzyme mediated aerobic composting of vegetable market complex waste (VMCW) have been investigated. Conventional composting technique though being capable of handling large quantum of waste are found to consume more time. Proven to be disadvantages factor. In the present investigation, the pre-cultured seed inoculums used for vegetable market complex waste, shortened the typical composting period from 45 to 9 days for the first time. Also, rapid size and volume reduction of VMCW was witnessed. The organic degradation of VMCW was observed as 42% (82 ± 2.83% to 40.82 ± 0.61%), with a volume reduction from 0.012m^3^ to 0.003 m^3^ within 9 days. An enriched nutrients NPK level of compost bio-fertilizer was recorded as 0.91% w/w, 0.5% w/w and 1.029% w/w respectively. Compost maturity observed through the X-ray diffraction (XRD) analysis of the manure confirmed the conversion of the crystal structure of the compost particle to amorphous form and the mineralization of organic matter during the composting. Thus, the fermented pre-cultured seed inoculums favored an enhanced nutrients level with shortened composting time.

## Introduction

India generates about 1,57,478 tons of municipal solid waste including vegetable market waste daily^1^. The capacity to treat the waste generated less than 20% and the remaining 80% waste is therefore dumped directly on to landfill without treatment. The municipal debris of Chennai corporation is dumped in Kodungaiyur (200 acres) and Perungudi (200 acres) dumpyard with the capacity of 2100- 2300 MT and 2200 to 2400 MT respectively. The unscientific dumping of waste in the landfills produced emissions of several greenhouse gases, majorly methane and carbon dioxide thereby contributing to global climate change^2^. To minimize the dumping of organic waste and thereby reducing greenhouse gas emission, several measures including composting are being experimented by investigated worldwide.

One of the main advantages of the biological aerobic composting method is that the organic waste compost conditions the soil and serves as fertilizers^3–10^. Researchers earlier observed that, as the VMCW gets degraded the size and volume reduce and the nutrients like nitrogen (N), phosphorus (P), potassium (K) get released to enrich the soil. Finstein et al.^11^, Kayhanian et al.^12^, Elving et al.^13^. studied the composting of such waste in exclusively designed and fabricated reactor. The addition of microorganisms and invertebrates to enhance various biochemical reactions yielding mature compost were investigated. It was reported that such composting not only reduced the net greenhouse gas emissions but also enhanced the carbon nitrogen ratio and soil productivity. The compost thus obtained proved to increase agricultural productivity, soil biodiversity and reduce the ecological risks, creating a better living environment for the micro-flora, fauna and humankind. It was also observed that the unwanted pathogens were destroyed and the volume of waste reduced, favoring land filling process.

Moisture content, temperature, C/N ratio, pH and coliform bacteria were chosen as parameters to control the quality, stability and efficiency of the composting process^8,14–18^. It has been reported that microbial decomposition of organic matter, occurs in the thin liquid (hygroscopic water) film around the surface of the particles enriched by water. During composting, the thin films of water surrounding individual particles are observed to dry off, making the microorganisms decomposing inorganic matter inactive. Thus, investigators concluded that the hygroscopic water around the waste particles to be a deciding factor in determining the efficiency of the composting process^19^. Temperature is reported to be a governing factor having an impact on the microbial activity during the composting process^20,21^. Temperatures below 20 °C and above 60 °C contribute in slowing down the composting process. Having observed the influence of high temperature and reduced moisture content on the efficiency of the composting^22,23^ incorporated aeration not only to have an optimal control on temperature and moisture content but also maintain the oxygen level^20,21,24,25^.

Traditional anaerobic and aerobic decomposition methods were reported to acieve mature compost in several months^26–28^. Recently developed techniques based on aerobic decomposition, reduced the composting period to about 4–5 weeks^29,30^. Further, reducing the time was attempted by few other researchers and concluded that seed inoculums could increase the microbial population, and produce desired enzymes enhancing the conversion of organics and reducing the odorous gas emissions^31,32^. However, less research on the enzyme activation and bio-kinetics modelling of aerobic composting are reported. Therefore, for the first time, an attempt has been made in formulating a precultured seed inoculum that can degrade VMCW by aerobic composting. To achieve size and volume reduction of VMCW by aerobic composting in a reduced time of seven days, a composter incorporated with shredding, aeration and mixing of VMCW has been designed, fabricated and used for the present study. This compact, easy to operate compacter for degradation of VMCW by aerobic composting is a novel attempt that not only reduces the size and volume of the waste but digest it in lesser time making the process viable. Further, the biokinetic modelling^33^ of the VMCW using the newly fabricated composter as also been investigated.

## Materials and methods

### Vegetable Market Complex Waste (VMCW)

The vegetable market complex waste was collected from Tambaram Municipality (Latitude: 12.9229°N, Longitude: 80.1275° E) located in Chennai, Tamil Nadu, India. It is reported that the solid waste generated from Tambaram Municipality requires 19.27 acres of landfill.

### Sample collection and shredding

Stratified random sampling technique was followed in order to eliminate the areas of non-uniform properties or concentrations which were identified and stratified. Thereafter, simple stratified random sampling technique was followed, so as to obtain a representative homogenous sample. The random sampling was done, where the vegetable market complex waste (VMCW) such as tomato, carrot, cabbage, radish, Ridge gourd, bottle gourd, brinjal, chayote, and beetroot were collected in mixed form and pooled further into a composite homogenized sample for the enhancement of composting processes.

### Mechanical shredder for VMCW breakdown

The VMCW shredding process was carried out in two stages. Primarily, the collected VMCW sample was broken into manageable pieces approximately 1.5 to 2 cc, which was then fed into a secondary shredder for further size reduction approximately to 0.5–0.6 cc.

### Aerobic Composting Reactor specifications

**Figure 1:**
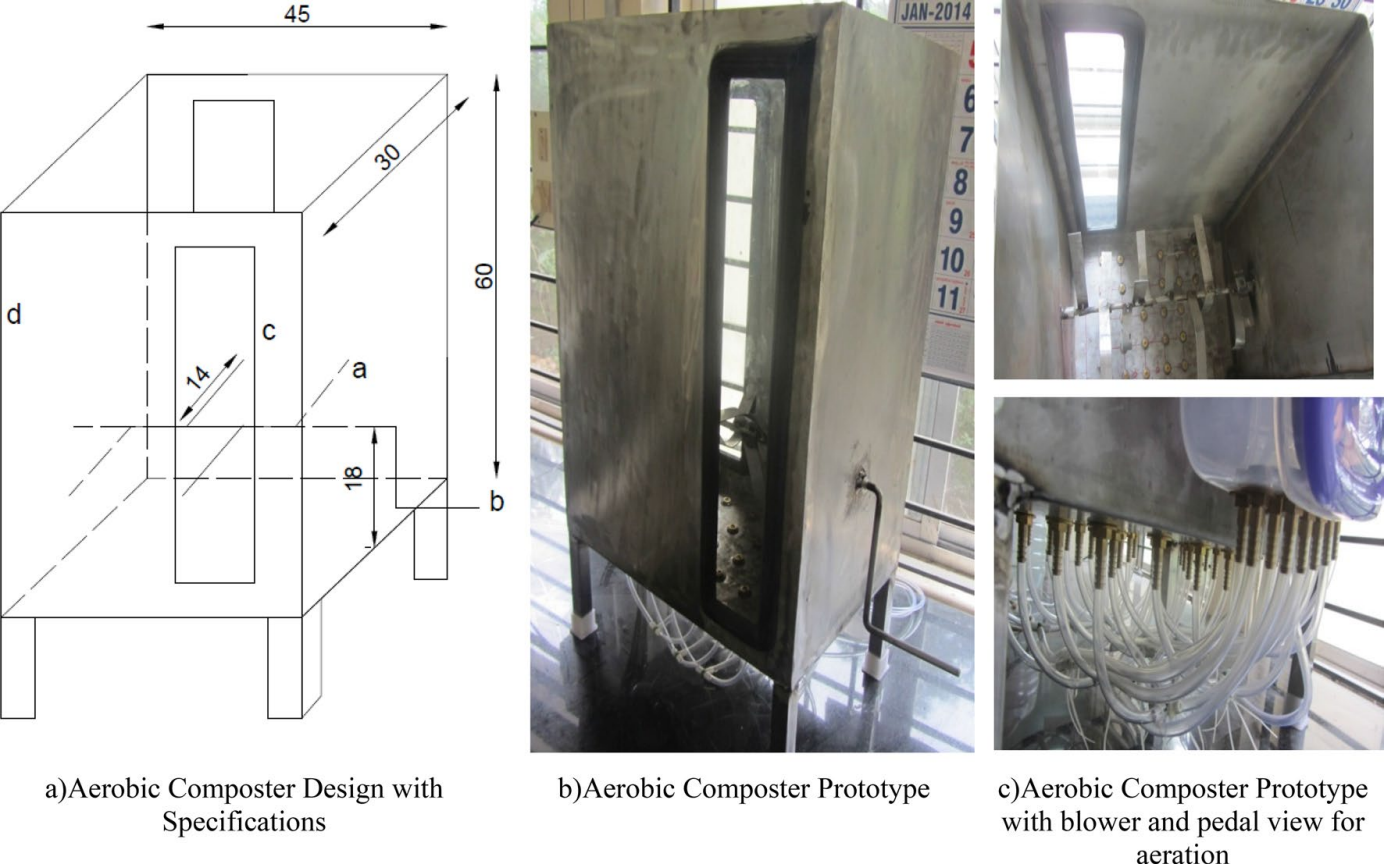
Aerobic composting setup.

The composting reactor prototype as per design depicted in Fig. 1a is made up of SS316, with the sizing of 45 × 30 × 60 in cm (L × B × H) and equipped with a manual stirring lever (Fig. 1b) with continuous aeration system through the air pump (capacity 230 V TID-75-P) (Fig. 1c) to maintain uniform homogenous mixing. The air enters the composting vessel (Fig. 1b) through the nozzle (Fig. 1c) and it was distributed through perforated holes in order to achieve uniform distribution of air throughout the vessel.

### Preparation and feeding of an enzyme into the composting reactor

An attempt has been made to prepare an enzyme (pre-cultured seed inoculum) using Jaggery and curd. Gabhane et al.^34^ have investigated the compost using different additives/catalyst and concluded that high microbial enzymatic activity, organic matter degradation, bulk density, quality of finished compost including gradation, were observed in the Jaggery additives. In addition to this, Partanen et al.^35^, have reported that the acceleration and the mature end product of composting was achieved by the addition of curd. The investigation on composting of municipal solid waste with jaggery and curd as seed inoculum was prepared by mixing 200 g of Jaggery and 10 g of curd with 1 L of water and pre-cultured for 5 days at room temperature under static condition. Bulking agent (rice husk) was added for increasing the surface area of the substrate which could increase the microbial activity^36^. Eventually, the bulking agent was sieved out at the end of the composting process which can be used as seed compost since it has a greater number of bacteria attached.

### Operation of aerobic bio composter

A sample of 12 kg (wet weight) Vegetable Market Complex Waste (VMCW) was taken and cut into small pieces of 0.5 cm in length using the mechanical shredder. The shredded vegetable market complex waste and seed inoculum with rice husk in the ratio of 16:1 was transferred into the composting reactor. The sample was collected on a daily basis at different depths before turning peddles and stored in a plastic container to monitor the composting process at room temperature. The parameters such as pH, TS, VS, volume reduction and temperature (at different depths) of the VMCW were noted at every 24 h.

### Characterization of Vegetable Market Complex waste (VMCW)

The VMCW used as substrates were characterized with total solids (TS) (APHA, 2540B), volatile solids (VS) (APHA, 2540G) and fixed solids (FS) (APHA, 2540E) using standard methods for the examination of water and wastewater^37^. The moisture content of the sample was measured after oven-dried at 105 °C for overnight^37^. The oven-dried sample was ground using pestle and mortar and then used for further analysis. The organic matter was calculated from the ash after igniting the sample (dry weight) in a muffle furnace at 550 °C for 2 h^37^. The pH of the compost was determined immediately upon collection of samples from the reactor by adding distilled water under the condition of solid to water ratio (1:10 w/v)^9^. The temperature in the bioreactor was measured manually during sample collection using a glass thermometer.

#### CHNS analysis

The elemental compositions such as carbon (C), hydrogen (H), nitrogen (N) and sulphur (S) of each organic waste were determined using CHNS analyzer (Model Euro vector 3000 series, Italy) (ASTM D-5291) available in CATERS CSIR-CLRI, Chennai, India and quantified based on the dry weight. The total and volatile solids were estimated using standard methods for the examination of water and wastewater^37^. An aliquot of the sample was oven-dried at 105 °C and kept in a desiccator prior to analysis then known weight of the dried VMCW was taken into an aluminium boat and the sample initial weights were noted. Thereafter, the boats were loaded in the CHNS analyzer and increased peak, the calibration graph was used to quantify the CHNS^38^. The oxygen content was calculated by the difference between VS and the total sum of C, N, H, S^39^. The calorific energy value (CEV) was estimated using Eq. (1) proposed by *Channiwala *et al.^40^.

1 $$ {\text{CEV}}\,({\text{MJ}}/{\text{kg}}) = 0.3491{\text{C}} + 1.1783{\text{H}} - 0.10340 - 0.0151{\text{N}} - 0.0211{\text{A}} $$

where, CHNO and A represents a percentage of carbon, hydrogen, nitrogen, oxygen and ash on dry weight basis.

#### XRD analysis

The overall structural changes during the decomposition of wastes were studied using X-Ray diffraction method. Prior to analysis, the sample was dried at 50 °C for 24 h and was ground to fine powder. The spectra were recorded on Bruker D8 Powder XRD instrument using the source Copper K alpha (CSIR-CLRI, Chennai).

### Microbial growth rate in the composter

1 g (wet weight) of composting sample was taken from the composting reactor at 24 h interval and serially diluted with 9 mL of sterile distilled water up to 10^−9^ dilution. Then 1 mL of the 10^−2^ to 10^−8^ the diluted sample was dispensed into a sterile petri dish with a molten Nutrient agar and gently swirls to solidify. The solidified plates were stored for 24–48 h. The microbial growth was expressed in CFU/g dry weight of total solids.

### Kinetics

Substrate concentration (S), and the consumption rate of the substrate (r) were observed during the composting period. The reciprocals of reaction rate (1/r) and Substrate concentration (1/S) were computed using a Lineweaver–Burk plot. In the Lineweaver-Burke plot, the intercept on the y-axis gives the value of K_3_, whereas the value of K_m_ is obtained from the slope of the line and the linear regression was obtained between 1/r and 1/S.

## Results and discussion

The aerobic composting was performed using novel pre-cultured seed inoculums in addition to the aeration and peddling system. The evaluation of the size and volume reduction with composting period revealed the effect of Jaggery and curd based pre-cultured inoculums which are detailed in the following sections.

### Characteristics of the VMCW

**Figure 2:**
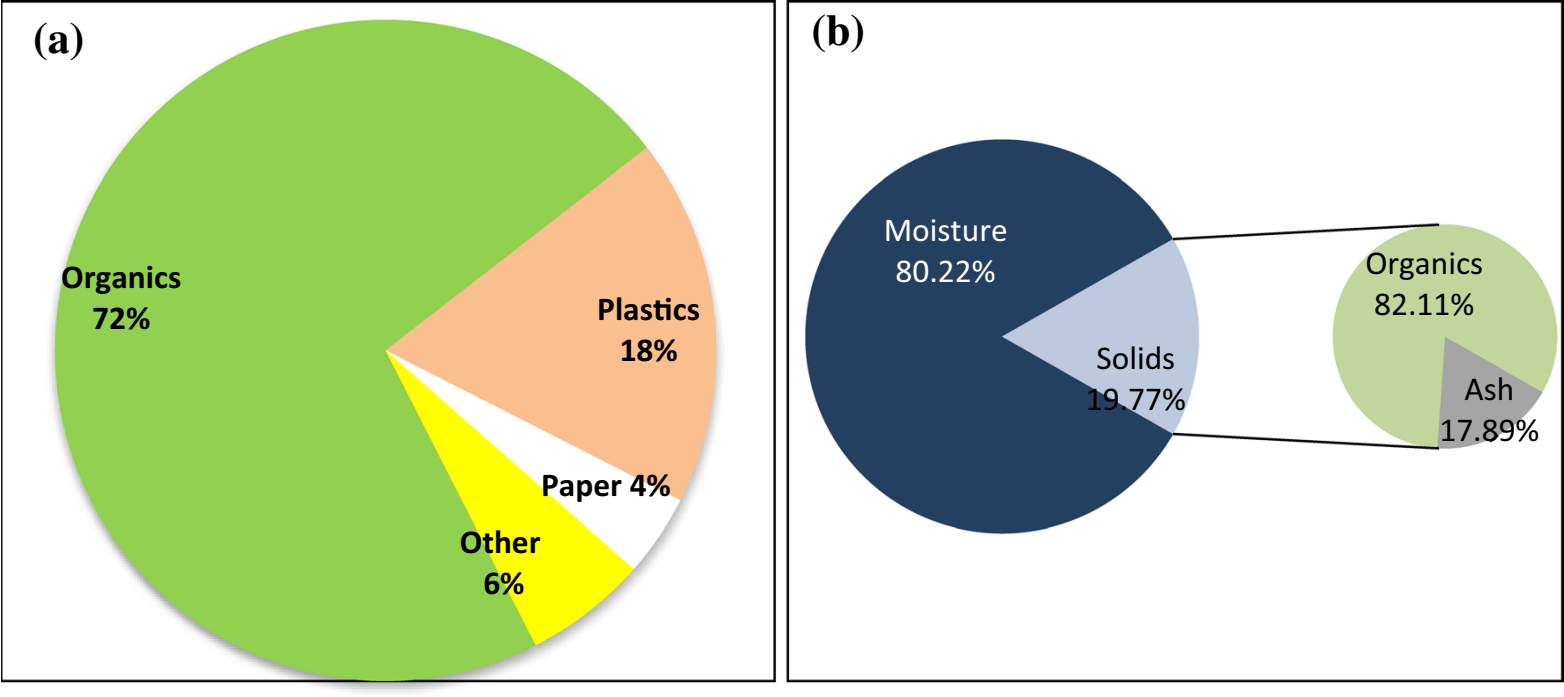
(**a**) Composition of VMCW at source. (**b**) Composition after sample collection.

The composition of VMCW collected from the Tambaram Municipality was quantified as 72% of organics, 18% of plastics, 4% of paper and 6% of miscellaneous from the complete source segregated, collected, weighed samples (Fig. 2a). Since it was separated at source, VMCW was taken for the aerobic composting operation which contains more than 80% organic waste (Fig. 2b) and the neutral pH of 6.8 was observed which favors microbial activity.

The initial total solids content was observed in the range of 19–22% (Fig. 2b). The initial volatile solid content observed was 82.11 ± 2.83% (Fig. 2b) illustrated a high fraction of organics in Vegetable Market Complex Waste consistent with results reported by Pellera et al.^41^. The empirical formula was calculated as reported as C_16_H_25_NO_4_ for the primary sedimentation tank sludge which indicates that the waste contains a lower value of degradable/non-degradable nitrogen content depicted higher C/N ratio at the end of the experiment (Table 1)^42^.

**Table 1:** Characterization of VMCW.

Parameter	Initial	Final
C/N	13.84	2.35
Empirical formula	C_16_H_25_NO_4_	C_10_H_17.9_ N
CEV (kJ/kg of TS)	25.05	19.45

### Effects of moisture and temperature

**Figure 3:**
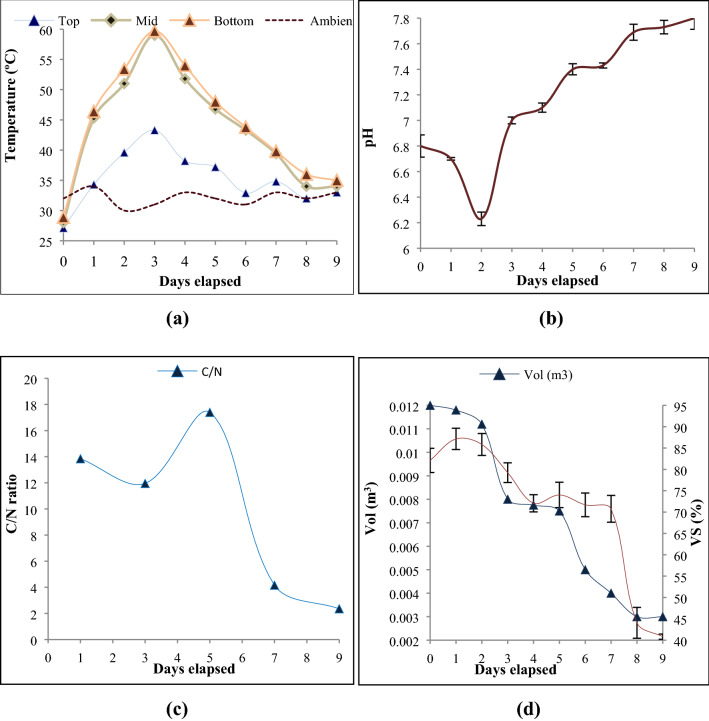
(**a**) Effect of temperature on composting, (**b**) effect of pH on composting, (**c**) effect of C/N ratio during the composting process, (**d**) evolution of volume reduction organic strength reduction and mineralisation.

Initial decomposition was carried out by mesophilic microorganisms thrived at 29.7 to 36 °C temperatures. Due to fine grinding of the VMCW, high moisture content (80.22 ± 0.34%) was observed initially, that has reduced the O_2_ uptake level and lowers the biodegradability potential of VMCW which was consistent with results reported by *Kalamdhad *et al.^43^^.^ In order to reduce the impact of moisture content, the bulking agent rice husk was mixed with VMCW, which further improved the surface area and dryness for microbial growth inside the reactor. Eventually, the matured compost with a reduced moisture content of 18.37 ± 0.29% (9th day) was observed at the end of the composting process (Fig. 3a)^44^.

**Figure 4:**
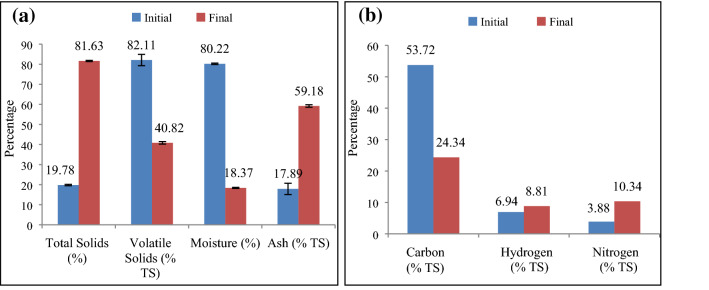
(**a**) VMCW at Initial and Final stage of composting, (**b**) CHN concentration at Initial and Final stage of composting.

The decreasing trend in moisture content observed was attributed to temperature and metabolic activity of VMCW, whereas the sample had high moisture content at the initial phase of composting reducing the oxygen supply. However, the reactor itself recovered from this drop of oxygen supply. The temperature was estimated at various depths (Fig. 4a) and every acuity achieved the estimation of 54 ± 9.27 on the third day, demonstrating that the heap had reached the thermophilic stage.The observed rapid reduction of moisture content (64%) on the 3rd day was attributed to the system acquired temperature by the metabolic enzymatic activity of microorganism for the protein degradation (Fig. 4a). This was confirmed with a decreased level of C/N ratio, degradation rate (VS 6.6%/ day) and reduction rate (volume reduction 3.2 L/day)^45–48^. Eventually, the temperature drop was observed at the final stage and, thereafter no reduction in temperature was observed attributed to insufficient bulk volume (3 L) and the liberation of excessive heat which indicates the completion of the aerobic composting process and production of matured manure.

### Effects of pH on composting

pH is the most influenced parameter in the solid waste biochemical metabolic reactions, which allows microorganisms to degrade the complex substances and increases the solubility of mineral elements during composting. The initial pH of 6.8 ± 0.08 (Fig. 3b) was observed for VMCW, enhance the growth of micro-organism could lead to high volume reduction, was supported by Khan et al.^49^, Zorpas et al.^50^. Further, the pH of the reactor was decreased to 6.23 on the 2nd day was attributed to the rapid particulate hydrolysation by high microbial of 2.9 × 10^−6^ CFU/g (Fig. 8) and enzymatic activity during the aerobic microbial metabolism in addition to the high temperature of 53 °C on 2nd day which increases the hydrogen ion concentration. Similarly, reports have been revealed that the disintegration of bigger sized substrate inhibits the oxygen supply, which could lead to anaerobic condition causes acid accumulation in the substrate that has reduced the pH. Subsequently, a gradual increase in pH to 7.8 was observed with days elapsed (3rd to 9th day) (Fig. 3b) which was attributed to proteinaceous degradation and formation of ammonia bicarbonate contributed to alkalinity which was prolonged up to the end of the experiment. This has favored adequate growth of micro-organism in moderately alkaline condition. Supporting to this, decreased pH up to 5.5 on 3rd day and thereafter increased pH of 8.5 up to end of the matured manure production period was observed by Gabhane et al.^34^.

### Effects of carbon–nitrogen ratio on composting

The maxima and minima C/N ratio were observed as 17.39 and 2.35 on 5th day and 9th day respectively (Fig. 3c) was attributed to the low and high complexity of carbohydrate and protein compound degradation. The study focused on the effect of pre-cultured seed inoculums on compost and its maturity (C/N ratio) (Fig. 3c). The C/N ratio was slightly reduced (11.95) during the thermophilic phase (3rd day) attributed to a high rate of degradation of carbohydrates where the aeration got disturbed due to the hydrolysation. This was in line with the study reported by Chai et al.^51^.

Consequently, slow degradation of protein compounds into ammonia reduces the nitrogen compound, thereby increasing trend of C/N ratio of 17.39 was observed up to 5th day which was corroborated well with high volume reduction (56%) (Fig. 3d) on the 5th day. This was consistent with results reported by Awasthi et al.^52^. Subsequently, the fall in the C/N ratio was observed could be regarded as being caused by microbial respiration due to the rapid uptake of available organic carbon by microorganisms. Thus, large amounts of CO_2_ were liberated leading concomitantly to an increased proportion of total nitrogen of the medium from 3.88% to 10.39% (Fig. 4b) which was consistent with the results of Karak et al.^53^, Awasthi et al.^52^. Eventually, the minimum carbon (24.35%) and maximum nitrogen (10.33%) on 9th day (Fig. 4b) was lower than the initial C/N ratio evidenced the perfect aerobic degradation and maturity of manure which was consistent with the maximum volume reduction of 77% (Fig. 3d)^54^.

### Volume and organic strength reduction in compost

The volumetric reduction of VMCW depends on the microbial degradation of the organic content available for composting reaction and moisture level inside the composter. In enzymatic pre-cultured seed inoculum-based composting, reduction in size of the material depends on the degree of hydrolysis of substrate by the bacteria replenishing enzymes which breakdown the complex polymeric organic materials into simpler ones. The volume of the compost material was decreased day by day with increased composting time (Fig. 3d), from 0.012 m^3^ on 0th day to 0.003 m^3^ on 9th day. The low initial rate of reduction in volume (0.0002 m^3^/day on 1^st^ day and 0.0006 m^3^/day on 2nd day) observed was attributed to the insufficient oxygen supply for aerobic microbes resulting from the high initial moisture in the substrate which reduces the air supply on the top and the middle surface of the composter. This was consistent with the results reported by Makan et al.^55^.

High volume reduction was recorded on 3rd day (0.0032 m^3^/day) which was attributed to the initial settlement of VMCW on the reactor due to reduced moisture content and further confirmed by the high microbial activity on 3rd day (3.6 × 10^−6^ CFU/g) (Fig. 8) which was consistent with volatile solids reduction beyond 3rd day. Thereafter, gradual volume reduction was observed with elapsed time. Eventually, no volume reduction was observed on 8th and 9th day of the composting period, as the composting is completely depending on the volume of voids in the waste and further, attainment of shrinkage limit of waste due to reduced moisture content along with converted matured manure didn’t have any effect on volume reduction. The decreased trend in VS was noticed from 2nd to 8th day (Fig. 3d) during the mesophilic and thermophilic phases of the composting and this change of the volatile solid content during composting was used to assess mineralization and decomposition of organic matter^56^. Eventually, the VS content reached relatively the stable form on 9th day which reveals that the substrate conversion had reached a stable phase and was matured. It was observed that, 82 ± 2.83% of TS was volatilised during the composting process and this loss contributed to the degradation of organic compounds (Table 2)^57^.

**Table 2:** Vegetable Market Complex Waste (VMCW) Composition.

Days elapsed	TS (% of waste)	VS (% of TS)	Ash (% of TS)	Moisture (% of waste)	C (% TS)	H (% TS)	N (% TS)	C/N ratio
0	19.78 ± 0.35	82.11 ± 2.83	17.89 ± 2.83	80.22 ± 0.35	53.72	6.94	3.88	13.84
3	35.89 ± 3.09	79.24 ± 2.31	20.76 ± 2.31	64.11 ± 3.09	73.00	5.44	6.10	11.95
5	45.71 ± 7.17	74.03 ± 2.96	25.97 ± 2.96	54.29 ± 7.17	74.62	12.01	4.29	17.39
7	55.89 ± 2.81	70.77 ± 3.13	29.23 ± 3.13	44.11 ± 3.13	37.18	5.50	4.34	4.16
9	81.63 ± 0.29	40.82 ± 0.62	59.18 ± 0.62	18.37 ± 0.29	24.35	8.81	10.94	2.35

**Figure 5:**
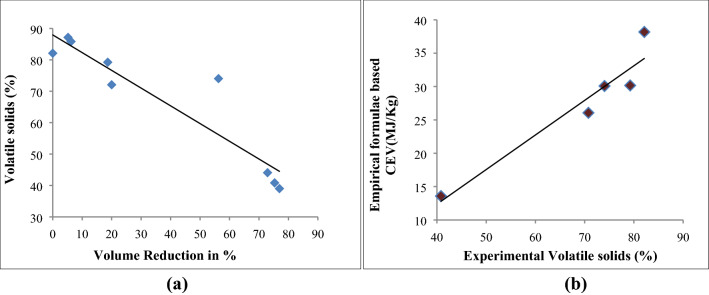
Linear relation between (**a**) VS and Volume reduction, (**b**) VS and CEV.

High correlation (R^2^ = 0.862) (Fig. 5a) between the percentage of volatile solids and percentage of volume reduction was observed for VMCW indicated that stabilization of organic content into manure which normally mineralized to nitrogen (N), phosphorus (P) and potassium (K) in addition to the other components with an organic mass in the volume of the composting reactor. The energy loss during the conversion of waste into manure was observed as 5600 kJ/kg of TS attributed to the decomposition of carbohydrates and protein compounds. Supporting to this the organic carbon decreased by 29.375% which was consistent with observed the volatile solids reduction of 40.6 ± 0.61%. This conforms the utilization of carbon for aerobic degradation and mineralization to mature compost was further confirmed with high correlation (R^2^ = 0.913) between volatile solids and CEV (Fig. 5b).

Moreover, the calorific energy value (CEV) of the manure was observed to be as 19,450 kJ/kg of DM evidenced the maturity and further confirmed by the reduction of moisture to less than 15% on 9th day. This was consistent with results reported by Stainforth et al.^58^ for wheat straw 17,600 kJ/kg. On the contrary, the maturation and stabilization pressure for the change of ammoniacal nitrogen to nitrate nitrogen and further conversion took 14 weeks, where the calorific energy value of the manure observed to be 10,000 kJ/kg DM with a moisture content of 12%are reported by Raclavska et al.^59^. This was in line with the results reported by many researchers and evaluated the CEV ranged from 7000 to 20,000 kJ/kg. For instance, the CEV of 8092 kJ/kg for bio-solids and wood chips was observed by Ekinci et al.^60^. Similarly, Steppa et al.^61^ reported for organic waste as 9000–11,000 kJ/kg, which was lower than that reported by Sobel and Muck^62^ for poultry droppings (12,800 kJ/kg), whereas the paper mill sludge and poultry manure compost reported CEV of 3649 kJ/kg, and for 5111 kJ/kg for straw and poultry manure compost^63^.

### XRD spectra of crystallised mature manure

**Figure 6:**
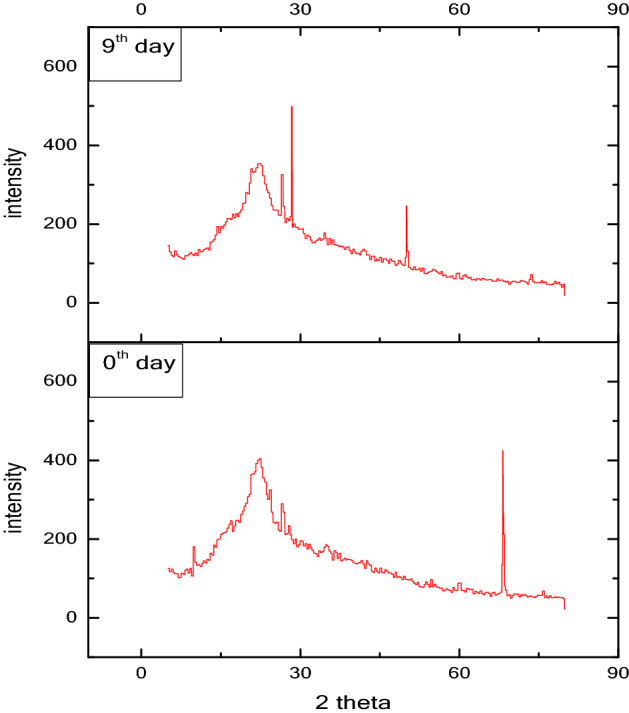
XRD spectra of VMCW compost.

Diffraction designs were contracted utilizing a goniometer. X-beam diffraction spectra of VMCW at various development stages (0th and 9th) as appeared in Fig. 6. It was seen that the quantity of exceptional peaks presents in spectra on 0th day test has diminished towards the 9th day of the treating the soil cycle, while less serious pinnacles expanded towards later phases of the treating the soil cycle.

This demonstrates the transformation of strong squanders from translucent to vague during the degradation cycle, showing that the cellulose and different polysaccharides mixes were corrupted. This was reliable with results for the humidification and developing cycle of the excrement straw detailed by Hu et al.^64^.

### Effects of the enzyme on the mineral nutrient of matured compost

**Figure 7:**
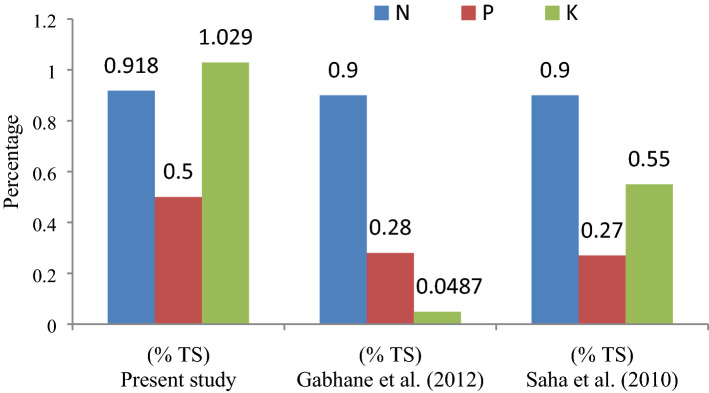
Comparison of NPK of present study with published results.

The concentration of mineral nutrients such as Nitrogen (N), Phosphorous (P) and Potassium (K) was observed as 0.918%, 0.5% and 1.029% respectively for the matured manure produced at reduced composting time of 9 days. It was observed that the NPK concentration in the obtained compost manure on 9th day was comparable with other literature (Fig. 7) reported the composting manure nutrients produced at 45–75 days. This reveals that the high activity of Jaggery based seed inoculums converts the high solid waste molecules into nutrients in the short time period of 9 days.

Supporting this, the N, P and K value was 0.9%, 0.28% and 0.0487% respectively and reported that the availability of these nutrients was high in Jaggery based enzyme when compared with other enzymes, which are based on fly ash, phosphor gypsum, lime, and polyethene glycol revealed by Gabhane et al.^34^. Similarly, value of N, P and K in the MSW compost varied from 0.9%, 0.27% and 0.55% respectively was addressed by Saha et al.^65^.

### Kinetic modelling of enriched microbial composting

**Figure 8:**
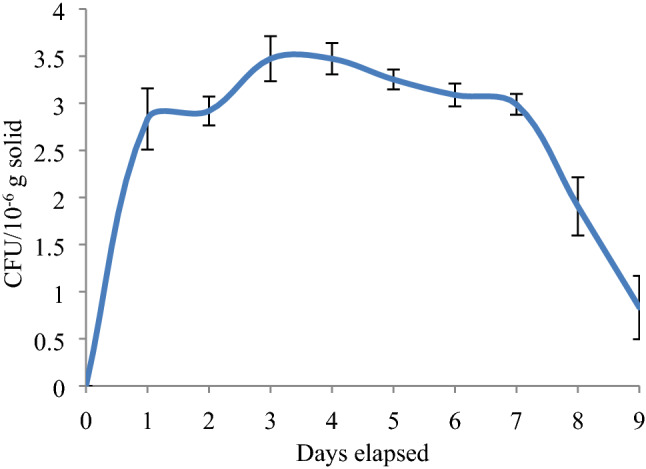
Microbial growth rate of composting process.

To analyze the effect of pre-cultured seed inoculums on VMCW composting process, the total microbial growth counts of the composting system were examined. The maximum microbial growth rate of 3.47 × 10^−6^ CFU/g was observed on 4th day (Fig. 8) whereas, in the normal composting process, the microbial consortia activity was delayed due to the acclimatization of the same to the vegetable waste which has increased the composting time. This could lead to odour production and maggot formation. The sharp lag phase observed on 2nd day was attributed to the high adaptability of microbes and the decomposition of the substrate which was confirmed by the acidic the pH of 6.2. Further, the exponential and declining microbial growth phases observed was evidenced in the short period of acclimatization and maturity (Fig. 8). In addition, the exponential growth phase was prominent on 3rd day and stationary phase was observed until the 5th day which reveals that the shortened stationary phase was attributed to efficient utilization of enzymes. Moreover, the microbial growth has coincided well with high VS, volume reduction and optimum temperature (Fig. 3a), pH (Fig. 3b) and C/N ratio (Fig. 3c). Declining phase depicted afterwards, where the microbial activity occurs on the thin films of water surrounded on the substrate. Thereafter, this hygroscopic water was dried so that the microbial activity was arrested at the end of the experiment, which was consistent with temperature (Fig. 3a), pH (Fig. 3b), C/N (Fig. 3c), Volume reduction (Fig. 3d).

The two stages of pre-cultured seed inoculum the composting process were based on microbial population for the former stage and substrate concentration for the later stage as a limiting factor. Initially, organic matter was sufficient to be decomposed where the microbial population was insufficient which limits the composting process. Thereafter, the microbial concentration was increased with decreased organic matter concentration which could lead to reduced heat due to low metabolic activity which was insufficient to maintain the composting temperature so that the substrate became the limiting factor for the composting process. The fundamental microbial kinetics were plotted and analysed was used to examine the efficiency of inoculation during the aerobic composting processes with reference to the Xi et al.^66^.

**Figure 9:**
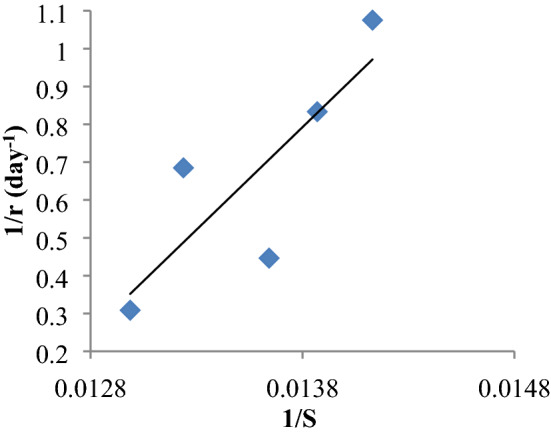
Determination of bio-kinetic constants.

The organic matter was plotted against composting time and the reaction rate (r) was determined by drawing the tangential resultant curve. The reciprocals of reaction rate (1/r) and Organic matter (1/S) were computed (Fig. 9). The Michaelis–Menten constant (K_m_) and limiting velocity reaction rate constant (K_3_) for the composting reactor was 81.06 and 0.15 respectively. The reaction rate (r) of the aerobic composting was very high especially on 8th day which was observed as 26.71%/day. This has depicted a high degradation potential of VMCW. Hence, the addition of enzymes proved to be attained rapid degradation of organic waste during composting under aerobic condition.

The Michaelis–Menten constant (K_m_) and limiting velocity reaction rate constant (K_3_) for the composting reactor was 81.06 and 0.15 respectively. The reaction rate (r) of the aerobic composting was very high especially on 8th day (26.71%/day), depicted a high degradation potential of VMCW. Hence, the addition of enzymes proved to be a rapid degradation of organic waste during composting under aerobic condition.

## Conclusion

The aerobic composting duration was considerably shortened to 9 days from the traditional composting period of 45 days, using a novel pre-cultured seed inoculum enzyme obtained from Jaggery and curd. The natural debasement of VMCW was 42% (82 ± 2.83% to 40.82 ± 0.61%), with a volume decrease from 0.012m3 to 0.003 m3 inside 9 days. An improved supplements NPK level of manure bio-compost was recorded as 0.91% w/w, 0.5% w/w and 1.029% w/w separately. This was evident with an increased microbial population by the plate count confirmed with a lag phase of microbial growth on 2nd day with an acidic pH, lower growth (CFU/mL). The shortened log phase on 5th day was attributed to an optimum temperature, C/N, pH coincided with high VS and volume reduction. The observed bio-kinetic constants based on the microbial count, substrate concentration was recorded as Km of 81.06, and K3 of 0.15 coincided with size and volume reduction. The enriched nutrients confirmed the reduced composting period, and this was further confirmed by the XRD analysis. Fertilizer development seen through the X-beam diffraction (XRD) examination of the excrement affirmed the transformation of the precious stone structure of the manure molecule to indistinct structure and the mineralization of natural issue during the treating of the soil. The lower K3 and improved microbial composition depicted an enhanced composting process in aid of pre-cultured bacteria/enzymes becoming a better alternative for VMCW degradation that can reduce the land requirement. In this way, the aged pre-refined seed inoculums supported an improved supplements level with abbreviated fertilizing soil time.

## Supplementary information

Supplementary Information.
